# Real-world treatment pattern and comprehensive comparative effectiveness of Endostar plus different chemotherapy in advanced patients with non-small cell lung cancer

**DOI:** 10.1038/s41598-022-14222-w

**Published:** 2022-06-27

**Authors:** Wei Jiang, Wei Sun, Wenhui Li, Jin Gao, Hui Wang, Wei Zhou, Jing Liang, Lixiang Aa, Luhua Wang

**Affiliations:** 1 Department of Radiation Oncology, National Cancer Center/National Clinical Research Center for Cancer/Cancer Hospital and Shenzhen Hospital, Chinese Academy of Medical Sciences and Peking Union Medical College, Shenzhen, 518116 Guangdong China; 2 Department of Thoracic Surgery, The Third Clinical College of Xinjiang Medical University, Urumqi, 830000 Xinjiang China; 3 Department of Radiation Oncology, Yunnan Cancer Hospital, Kunming, 650106 Yunnan China; 4 Department of Radiation Oncology, Anhui Provincial Hospital, Hefei, 230031 Anhui China; 5 Department of Radiation Oncology, Hunan Cancer Hospital, Changsha, 410013 Hunan China; 6 Department of Radiation Oncology, Chongqing Cancer Hospital, Chongqing, 400030 China; 7 Department of Radiation Oncology, Shaanxi Provincial Cancer Hospital, Xi’an, 710061 Shaanxi China; 8 The State Key Laboratory of Translational Medicine and Innovative Drug Development, Nanjing, 210042 Jiangsu China

**Keywords:** Lung cancer, Targeted therapies

## Abstract

Recombinant human endostatin (Endostar) plus vinorelbine/cisplatin (NP) had been approved for the treatment of non-small cell lung cancers (NSCLC). But the real-world treatment pattern and effectiveness of Endostar plus other combination chemotherapy, namely docetaxel/platinum (DP), gemcitabine/platinum (GP), pemetrexed/platinum (PP), and paclitaxel/platinum (TP) in both treatment-naïve and re-treatment patients with advanced NSCLC were still unclear. A retrospective observational study was conducted based on the electronic medical record (EMR) system and advanced patients with NSCLC were identified from 7 cancer hospitals in China from 2012 to 2019. These patients were divided into five groups, Endostar plus NP, Endostar plus DP, Endostar plus GP, Endostar plus PP, and Endostar plus TP groups. The disease control rate (DCR), overall response rate (ORR), and the progression-free survival (PFS) were evaluated. Of the eligible 512 advanced patients with NSCLC, 10.35% were in Endostar plus NP group, while the numbers were 15.43%, 32.42%, 26.56%, 15.23% in Endostar plus DP group, Endostar plus GP group, Endostar plus PP group, and Endostar plus TP group, respectively. The ORRs were 31%, 28%, 22%, 41% and 27%, and the DCRs were 71%, 72%, 57%, 72% and 76%, respectively. The median of PFSs for the above groups were 7.9, 6.8, 5.6, 13.7, and 5.4 months. Compared with Endostar plus NP group, the hazard ratios (HRs) and 95%CIs of Endostar plus other chemotherapy were 1.86 (0.75–4.61), 2.15 (0.83–5.60), 1.33 (0.51–3.44), and 2.42 (0.86–6.81). This real-world study found the effectiveness of Endostar plus DP, Endostar plus GP, Endostar plus PP, and Endostar plus TP were of no statistically significant differences compared with Endostar plus NP and reflected the good effectiveness of Endostar plus different chemotherapy in advanced patients with NSCLC.

## Introduction

Lung cancer remains a great global health burden as the deadliest cancer with an estimated 2,206,771 new cases diagnosed and 1,796,144 deaths in 2020 worldwide, and 787,000 new cases diagnosed and 631,000 deaths in China since 2015^1,2^. Non-small cell lung cancers (NSCLC) comprise about 85% of all lung cancer, and the majority are diagnosed in late stage^3^.

Platinum-based chemotherapy remains the primary first-line treatment for the advanced NSCLC patients, but the efficacy is unsatisfactory^4^. Thus, a new strategy, such as antiangiogenic therapy and immunotherapy, for NSCLC therapy is urgent. Recombinant human endostatin (Endostar), an angiogenesis inhibitor, has shown the effect of downregulating transplantation matrix metalloproteinases (MMP) and vascular endothelial growth factor (VEGF) to inhibit neovascularization and tumor growth^5^. Endostar has also shown clinical efficacy in the treatment of advanced NSCLC in China^6–8^.

Although clinical application of antiangiogenic therapy has brought promise for the treatment of NSCLC and Endostar plus vinorelbine/cisplatin (NP) were approved by the Chinese Food and Drug Administration (CFDA) in 2005, in real-world clinical practice, NSCLC patients are treated with different regimens according to their physical and economic status. Although studies were conducted focused on first-line treatment patients or early-stage patients, the real-world treatment pattern and evidence of the effectiveness of Endostar in combination with other chemotherapy regimens in advanced NSCLC patients remains unclear and need to be explored. Therefore, this study aimed to evaluate the treatment pattern and effectiveness of Endostar plus different combination chemotherapy, such as Endostar plus docetaxel/platinum (DP), Endostar plus gemcitabine/platinum (GP), Endostar plus pemetrexed/platinum (PP) and Endostar plus paclitaxel/platinum (TP) compared with Endostar plus NP in treatment-naïve patients and re-treatment NSCLC patients with advanced-stage in real-world settings.

## Results

### Real-world treatment pattern and baseline characteristics

Table 1 showed the real-world treatment pattern and baseline characteristics of Endostar plus different chemotherapy in all 512 patients with advanced NSCLC. Endostar plus NP group only accounted for 10.35%, while Endostar plus DP group accounted for 15.43%, Endostar plus GP group accounted for 32.42%, Endostar plus PP group accounted for 26.56%, and Endostar plus TP group accounted for 15.23%. The mean ages were 57.0, 57.9, 59.2, 56.4 and 59.3 years old, and male patients accounted for 66.0%, 69.6%, 88.0%, 63.2% and 80.8% respectively. The real-world treatment pattern and baseline characteristics of treatment-naïve patients and re-treatment patients were summarized in Table 2.

**Table 1:** Real-world treatment pattern and baseline characteristics of Endostar plus different chemotherapy in all patients.

Variables	All patients	Endostar plus NP	Endostar plus DP	Endostar plus GP	Endostar plus PP	Endostar plus TP
N (%)	512	53 (10.35)	79 (15.43)	166 (32.42)	136 (26.56)	78 (15.23)
**Age, years**						
Mean ± SD	58.1 ± 10.0	57.0 ± 8.2	57.9 ± 9.6	59.2 ± 9.3	56.4 ± 11.7	59.3 ± 9.3
Median (Q1-Q3)	59.2 (51.3–65.0)	56.1 (51.0–62.0)	58.1 (50.3–65.6)	60.1 (53.5–65.7)	58.6 (48.0–65.0)	59.6 (54.1–65.4)
**Sex, n (%)**						
Unknown	14 (2.7)	2 (3.8)	2 (2.5)	5 (3.0)	1 (0.7)	4 (5.1)
Male	385 (75.2)	35 (66.0)	55 (69.6)	146 (88.0)	86 (63.2)	63 (80.8)
Female	113 (22.1)	16 (30.2)	22 (27.8)	15 (9.0)	49 (36.0)	11 (14.1)
**Smoking history**						
No	185 (36.1)	24 (45.3)	31 (39.2)	44 (26.5)	70 (51.5)	16 (20.5)
Yes	327 (63.9)	29 (54.7)	48 (60.8)	122 (73.5)	66 (48.5)	62 (79.5)
**Family history of lung cancer**						
No	493 (96.3)	51 (96.2)	77 (97.5)	163 (98.2)	131 (96.3)	71 (91.0)
Yes	19 (3.7)	2 (3.8)	2 (2.5)	3 (1.8)	5 (3.7)	7 (9.0)
**Disease stage**						
III	188 (36.7)	21 (39.6)	33 (41.8)	83 (50.0)	21 (15.4)	30 (38.5)
IV	324 (63.3)	32 (60.4)	46 (58.2)	83 (50.0)	115 (84.6)	48 (61.5)
**Pathological type**						
Squamous cell carcinoma	181 (35.4)	20 (37.7)	36 (45.6)	99 (59.6)	6 (4.4)	20 (25.6)
Adenocarcinoma	205 (40.0)	19 (35.8)	37 (46.8)	8 (4.8)	116 (85.3)	25 (32.1)
Unknown	118 (23.0)	12 (22.6)	3 (3.8)	59 (35.5)	12 (8.8)	32 (41.0)
Other	8 (1.6)	2 (3.8)	3 (3.8)	0 (0)	2 (1.5)	1 (1.3)
**Patient type**						
Treatment-naïve	417 (81.4)	26 (49.1)	54 (68.4)	148 (89.2)	122 (89.7)	67 (85.9)
Re-treatment	95 (18.6)	27 (50.9)	25 (31.6)	18 (10.8)	14 (10.3)	11 (14.1)

**Table 2:** Real-world treatment pattern and baseline characteristics of Endostar plus different chemotherapy in treatment-naïve and re-treatment patients.

Variables	Treatment-naï ve patients (N = 417)					Re-treatment patients (N = 95)				
	Endostar plus NP	Endostar plus DP	Endostar plus GP	Endostar plus PP	Endostar plus TP	Endostar plus NP	Endostar plus DP	Endostar plus GP	Endostar plus PP	Endostar plus TP
N (%)	26 (6.24)	54 (12.95)	148 (35.49)	122 (29.26)	67 (16.07)	27 (28.42)	25 (26.32)	18 (18.95)	14 (14.74)	11 (11.58)
**Age, years**										
Mean ± SD	57.2 ± 7.0	57.8 ± 10.2	59.5 ± 9.5	56.4 ± 11.6	59.8 ± 9.2	56.8 ± 9.3	58.0 ± 8.4	57.5 ± 7.0	56.9 ± 12.2	57.4 ± 9.6
Median (Q1-Q3)	57.2 (52.4–61.4)	58.9 (49.4–66.5)	50.7 (53.6–66.7)	56.4 (49.4–66.5)	58.9 (49.4–66.5)	55.0 (48.8–52.6)	58.0 (52.4–64.3)	57.6 (52.8–59.1)	57.8 (45.4–63.1)	56.5 (49.8–55.4)
**Sex, n (%)**										
Unknown	2 (7.7)	2 (3.7)	5 (3.4)	1 (0.8)	4 (6.0)	0 (0)	0 (0)	0 (0)	0 (0)	0 (0)
Male	17 (65.4)	38 (70.4)	131 (88.5)	76 (62.3)	54 (80.6)	18 (66.7)	17 (68.0)	15 (83.3)	10 (71.4)	9 (81.8)
Female	7 (26.9)	14 (25.9)	12 (8.1)	45 (36.9)	9 (13.4)	9 (33.3)	8 (32.0)	3 (16.7)	4 (28.6)	2 (18.2)
**Smoking history**										
No	10 (38.5)	22 (40.7)	38 (25.7)	63 (51.6)	12 (17.9)	14 (51.9)	9 (36.0)	6 (33.3)	7 (50.0)	4 (36.4)
Yes	16 (61.5)	32 (59.3)	110 (74.3)	59 (48.4)	55 (82.1)	13 (48.2)	16 (64.0)	12 (66.7)	7 (50.0)	7 (63.6)
**Family history of lung cancer**										
No	26 (100.0)	54 (100.0)	145 (98.0)	117 (95.9)	61 (91.0)	25 (92.6)	23 (92.0)	18 (100.0)	14 (100.0)	10 (90.9)
Yes	0	0	3 (2.0)	5 (4.1)	6 (9.0)	2 (7.4)	2 (8.0)	0	0	1 (9.1)
**Disease stage**										
III	12 (46.2)	21 (38.9)	75 (50.7)	17 (13.9)	28 (41.8)	9 (33.3)	12 (48.0)	8 (44.4)	4 (28.6)	2 (18.2)
IV	14 (53.9)	33 (61.1)	73 (49.3)	105 (86.1)	39 (58.2)	18 (66.7)	13 (52.0)	10 (55.6)	10 (71.4)	9 (81.8)
**Pathological type**										
Squamous cell carcinoma	8 (30.8)	23 (42.6)	88 (59.5)	3 (2.5)	18 (26.9)	12 (44.4)	13 (52.0)	11 (61.1)	3 (21.4)	2 (18.2)
Adenocarcinoma	5 (19.2)	27 (50.0)	5 (3.4)	107 (87.7)	18 (26.9)	14 (51.9)	10 (40.0)	3 (16.7)	9 (64.3)	7 (63.6)
Unknown	11 (42.3)	1 (1.9)	55 (37.2)	11 (9.0)	31 (46.3)	1 (3.7)	2 (8.0)	4 (22.2)	1 (7.1)	1 (9.1)
Other	2 (7.7)	3 (5.6)	0 (0)	1 (0.8)	0 (0)	0 (0)	0 (0)	0 (0)	1 (7.1)	1 (9.1)

### Effectiveness of Endostar plus different chemotherapy

292 patients who had at least one dosage of Endostar and at least one time of response evaluation record before and after baseline were included in the effectiveness analysis set with 25 in Endostar plus NP group, 49 in Endostar plus DP group, 90 in Endostar plus GP group, 89 in Endostar plus PP group and 39 in Endostar plus TP group.

**Figure 1:**
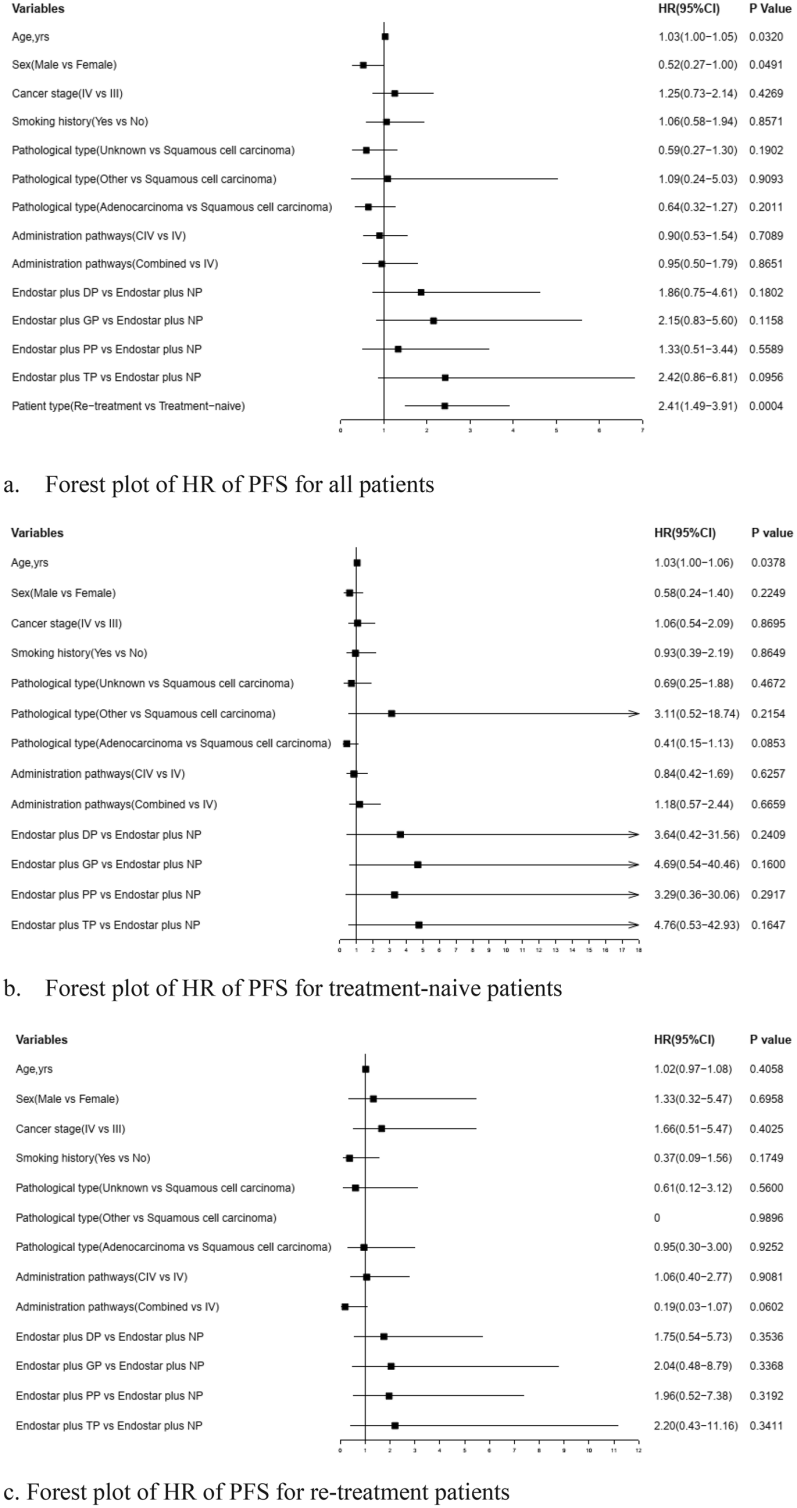
Forest plot of HR of PFS for all patients and subgroup patients.

Table 3 showed the overall response rates (ORRs) for the above five groups were 28%, 22%, 41%, 27% and 31%, and the disease control rates (DCRs) were 72%, 57%, 72%, 76% and 74%, and the median of progression-free survivals (PFSs) were 7.9, 6.8, 5.6, 13.7 and 5.4 months, respectively. These differences of DCRs, ORRs and PFSs in Endostar plus different chemotherapy regimens groups were not statistically significant. Table 4 reported the DCRs, ORRs and PFSs of Endostar plus different chemotherapy in treatment-naïve patients and in re-treatment patients, and there were also no statistically significant differences. The Cox regression model revealed there were also no statistically significant differences in PFSs of Endostar plus other chemotherapy compared with Endostar plus NP group after adjusting age, sex, smoking history, family history of lung cancer, disease stage, pathological type and administration pathways. Figure 1 showed that the hazard ratios (HRs) (95%CI) of Endostar plus other chemotherapy were 1.86 (0.75–4.61), 2.15 (0.83–5.60), 1.33 (0.51–3.44) and 2.42 (0.86–6.81) as compared with Endostar plus NP group, and the re-treatment patients had decreased PFS as compared with treatment-naïve patients (HR = 0.84 [95% CI 0.75–0.95]). The exploratory subgroup analysis also demonstrated the PFSs of Endostar plus different chemotherapy groups were of no statistically significant differences, no matter in treatment-naïve patients or in re-treatment patients (Fig. 1).

**Table 3:** The ORRs, DCRs and PFSs of Endostar plus different chemotherapy in all patients.

Variables	All patients	Endostar plus NP	Endostar plus DP	Endostar plus GP	Endostar plus PP	Endostar plus TP	*P* value
N	292	25	49	90	89	39	
ORR (%)	31	28	22	41	27	31	0.3667
DCR (%)	71	72	57	72	76	74	0.4104
Median PFS (95%CI), months	8.2 (6.0–13.7)	7.9*	6.8 (4.1–14.9)	5.6 (3.7–8.3)	13.7*	5.4*	0.1198

*Too few people to estimate confidence intervals.

**Table 4:** The ORRs, DCRs and PFSs of Endostar plus different chemotherapy in treatment-naïve patients and in re-treatment patients.

Variables	Subgroups in total	Endostar plus NP	Endostar plus DP	Endostar plus GP	Endostar plus PP	Endostar plus TP	*P* value
**Treatment-naïve patients**							
N	233	10	33	80	80	30	
ORR (%)	35	35	60	27	44	28	0.0813
DCR (%)	77	76	90	64	76	80	0.3045
Median PFS (95%CI), months	12.9 (7.2–14.9)	–*	14.9 (4.1–22.4)	5.6 (4.3–8.3)	16.0*	5.4*	0.2025
**Re-treatment patients**							
N	59	15	16	10	9	9	
ORR (%)	15	7	13	20	22	22	0.7725
DCR (%)	49	60	44	40	44	56	0.1886
Median PFS (95%CI), months	4.5 (2.6–9.0)	5.6 (2.6–7.9)	4.5 (1.8–9.7)	3.4 (0.0–17.4)	1.5*	5.8 (0.0–5.8)	0.8230

*Too few people to estimate PFS and confidence intervals.

## Discussion

Endostar is an effective angiogenesis inhibitor which can directly target new capillary endothelial cells around the tumor^9,10^. Study showed that median PFS in the Endostar plus NP regimen was significantly increased compared with NP alone (6.3 and 3.6 months, *P* < 0.001), as well as the ORR (35.4% and 19.5%, *P* < 0.01)^8^. Endostar was approved in combination with NP for the treatment of NSCLC by the Chinese Food and Drug Administration in 2005, but the treatment pattern and real-world evidence of different combined regimens were still unknown for both treatment-naïve and re-treatment NSCLC patients with advanced stage. This study found that Endostar plus GP, instead of Endostar plus NP which had been approved by the CFDA, was the domain combination regimens, especially in treatment-naïve patients, and the effectiveness of Endostar plus DP, Endostar plus GP, Endostar plus PP and Endostar plus TP weren’t statistically significant different compared with that of Endostar plus NP.

Recently, several studies had been performed to evaluate the effectiveness of Endostar combined with docetaxel chemotherapy. For patients with stage Ib–IIIa postoperative NSCLC, QI Daliang etc. found that the PFS of Endostar plus docetaxel and carboplatin was not statistically significant than that of docetaxel and carboplatin alone^11^. But our study found, in the advanced patients with NSCLC, there was also no statistically significant effectiveness difference in Endostar plus NP group and Endostar plus DP group. The different result may be due to different TNM stages, and this study focused on advanced-stage patients represented patients with systematic treatment.

The better effectiveness of Endostar plus GP had been certified by several studies compared with GP alone in small samples of patients with NSCLC^12,13^. For example, in a study including 49 patients with advanced NSCLC, Yu Xun etc. found that the PFS in Endostar combined with gemcitabine and carboplatin regimen group was longer than that in gemcitabine and carboplatin regimen group with a statistically significant difference (*P* < 0.05)^12^. This study included 512 NSCLC patients with advanced stage and showed the PFS of Endostar plus GP was not significantly different from Endostar plus NP group. To some extent it can be said that this study demonstrated the good effectiveness of Endostar plus GP in a large sample patient.

For the efficacy of pemetrexed/cisplatin (PC), a trend of prolonged PFS was found in 56 previously untreated patients with lung adenocarcinoma, although the difference was lack of statistical significance in Endostar plus PC group compared with PC alone group. While this real-world study revealed the effectiveness in 136 patients treated with Endostar plus PP was no statistically significant difference compared with Endostar plus NP group. The different results may be due to different pathological tissue typing. For non-squamous advanced NSCLC, pemetrexed/cisplatin still was a relatively modern regimen which needs to be further studied^14^.

Paclitaxel-carboplatin (TC) approved by US Food and Drug administration was the first-line treatment for NSCLC. In this real-world study, the PFS of Endostar plus TP group had no statistically significant difference compared with that of Endostar plus NP group. Similarly, Ma Huifang etc. also revealed the Endostar plus TP had better curative effect than TP alone in the lung adenocarcinoma patients with stage IIIb and IV^15^. While Han Baohui etc. found in previously untreated, advanced NSCLC patients, first-line treatment with Endostar plus TC seemed to have an increased ORR compared with TC alone, but the differences in PFS or OS between the two groups were not statistically significant^16^. The contradictory findings about Endostar plus TP might be due to different patient types such as treatment-naïve patients and re-treatment patients.

Besides, the study has several advantages. Above all, this study firstly explored the real-world treatment pattern both in treatment-naïve patients and re-treatment patients and found that Endostar plus NP approved by the CFDA wasn’t the main combination regimens. Secondly, the patients in this study were more representative, including advanced-stage NSCLC patients with different pathological tissue typing and re-treatment patients. Lastly, this study was conducted in a larger sample than previous studies and to some extent, the effectiveness analysis result without statistically significance provided real-world evidence for the expansion of the combination regimen.

We acknowledge this study still has two limitations. Firstly, just as all the real-world studies, the retrospectively collected EMR information might lead to bias, but in some extent, the bias was reduced because this study identified patients from 7 cancer centers in China and the confounding factors were adjusted by Cox regression model. Secondly, the sample size of re-treatment patients was smaller than that of treatment-naïve patients, although this study was conducted in a larger sample patient compared with previous study.

In conclusion, this retrospective multi-center study showed in real-world practice, the Endostar plus GP was the domain combination regimens in advanced-stage NSCLC patients and revealed that Endostar plus other chemotherapy regimens also had good clinical benefits, with relatively high ORRs and DCRs.

## Methods

### Patients

A retrospective multi-center observational study was conducted based on electronical medical records (EMR) from 7 cancer hospitals in China, registered on Chinese Clinical Trial Registry (ChiCTR2000035129). This study was approved and waived informed constent by all the institutional and licensing committee [Ethics committee of National Cancer Center/National Clinical Research Center for Cancer/Cancer Hospital & Shenzhen Hospital, Chinese Academy of Medical Sciences and Peking Union Medical College, Shenzhen, China (14/01/2020), Ethics committee of The Third Clinical College of Xinjiang Medical University, Urumqi, China (22/06/2020), Ethics committee of Yunnan Cancer Hospital, Kunming, China (29/03/2021), Ethics committee of Anhui Provincial Hospital, Hefei, China (10/06/2020), Ethics committee of Hunan Cancer Hospital, Changsha, China (10/03/2021), Ethics committee of Chongqing Cancer Hospital, Chongqing, China (22/05/2020), and Ethics committee of Shaanxi Provincial Cancer Hospital, Xi’an, China (25/03/2021)]. All research was performed in accordance with the the Declaration of Helsinki. Patients aged older than 18 years with clinical diagnosis of NSCLC, pathological stage III or IV [defined by American Joint Committee on Cancer tumor, node, metastasis (TNM) staging system version 7.0], and treated with Endostar combined with chemotherapy between 2012 and 2019 were included. According to the combination chemotherapy, the patients were furtherly divided into Endostar plus NP group, Endostar plus DP group, Endostar plus GP group, Endostar plus PP group and Endostar plus TP group. Treatment-naïve patients and re-treatment patients were separately defined as those who had Endostar administration during the 1st line therapy and those who had Endostar administration during 2nd line therapy or after, and exploratory subgroup analysis was furtherly performed.

### Baseline covariates

Index date was defined as the date of first treatment with Endostar, and the baseline covariates data were collected, including birth date, sex, smoking history, family history of lung cancer, admission date and discharge date, tumor stage, pathological feature and administration pathways. The smoking history, family history of lung cancer, tumor stage and pathological feature were extracted by Natural Language Processing (NLP) from admission records and discharge note in EMR.

### Effectiveness evaluation

The Response Evaluation Criteria in Solid Tumors (RECIST) Version 1.1 is used to evaluate the tumor response. ORR was defined as the percentage of patients who had complete response (CR) and/or partial response (PR). DCR was defined as the percentage of patients who had CR and/or PR and/or stable disease (SD). The PFS was defined as the interval(months) between first date of Endostar administration to the date of disease progression or death. In patients with progressive disease or death, the date of imaging progression or death was used as the endpoint date, and if PD and death were not observed, the date of the last evaluation was considered as the censored date.

### Statistical methods

SAS 9.4 (SAS Institute INC., Cary, NC) was used to describe patient characteristics and compare the effectiveness among different treatment regimens. Quantitative variables, age, was described using Mean ± SD and Median(P25-P75), and qualitative variables, sex, tumor stage, family history of lung cancer, smoking history, administration pathways, and treatment regimen were described as count and component ratio. The Chi-squared test or Fisher exact test was used to compare ORRs and DCRs. The Kaplan–Meier method was used to describe PFSs and log-rank test was used to compare the differences in different groups. The HR (95%CI) of different chemotherapy groups were estimated by Cox regression model. A two-sided *P* < 0.05 was considered statistically significant.

### Ethics declarations and consent to participate

This study is a retrospective, non-interventional study, which does not interfere with routine diagnosis and treatment, does not affect any medical rights of patients, does not increase the medical risk of patients. At the same time, the study did not identify individual patients. In addition, most of the patients to be included in this study have died or lost to follow-up and their informed consents could not be obtained. For the above reasons, we applied for exempting informed consents of all patients in this study and was approved by ethics committees from all centers. All research was performed in accordance with the Declaration of Helsinki.

The full name of all ethics committee that approved this study:

- Ethics committee of National Cancer Center/National Clinical Research Center for Cancer/Cancer Hospital & Shenzhen Hospital, Chinese Academy of Medical Sciences and Peking Union Medical College, Shenzhen, China (14/01/2020).
- Ethics committee of The Third Clinical College of Xinjiang Medical University, Urumqi, China (22/06/2020).
- Ethics committee of Yunnan Cancer Hospital, Kunming, China (29/03/2021).
- Ethics committee of Anhui Provincial Hospital, Hefei, China (10/06/2020).
- Ethics committee of Hunan Cancer Hospital, Changsha, China (10/03/2021).
- Ethics committee of Chongqing Cancer Hospital, Chongqing, China (22/05/2020).
- Ethics committee of Shaanxi Provincial Cancer Hospital, Xi’an, China (25/03/2021).

## Data Availability

The data of this study are available on request from the corresponding author [LW]. The data are not publicly available due to [state restrictions, “Where the information on human genetic resources of China is provided or opened for use to organizations, individuals or institutions established or actually controlled outside the country, it shall report in advance to the competent department of science and technology under The State Council and submit a copy of the information”. Adopted at the 22nd Session of the Standing Committee of the 13th National People’s Congress of the People’s Republic of China on October 17, 2020 and effective as of April 15, 2021].
